# Association Between Socioeconomic Status and Prevalence of Cardio-Metabolic Risk Factors: A Cross-Sectional Study on Residents in North China

**DOI:** 10.3389/fcvm.2022.698895

**Published:** 2022-03-07

**Authors:** Zhihua Hao, Mian Wang, Qiuxiao Zhu, Jie Li, Zibo Liu, Lingling Yuan, Yue Zhang, Lihui Zhang

**Affiliations:** Department of Endocrinology, Second Hospital of Hebei Medical University, Shijiazhuang, China

**Keywords:** socioeconomic status, cardio-metabolic risk factors, metabolic syndrome, public health, epidemiology

## Abstract

Studies have found associations between cardio-metabolic disorders and socioeconomic status (SES) in developed areas. However, little epidemiological data are available on residents of less developed areas in North China. A cross-sectional study that consisted of 2,650 adults randomly selected from local residents was conducted on a developing province, Hebei. SES was assessed in terms of education, personal income per year, and occupation. The association between SES and metabolic syndrome (MetS) was determined by multivariate logistic regression. The weighted prevalence of MetS was 26.8% among residents of Hebei province. The lower prevalence of MetS and abdominal obesity was associated with increase in SES groups. After adjustments regarding age, sex, body mass index, living area, smoking, salt intake, and family history of diabetes, odds ratio (OR) for elevated blood pressure (BP) of individuals with higher SES level was 0.71 [95% confidence interval (CI): 0.542–0.921] compared with those with lower SES level. Cardio-metabolic risk factors were commonly identified among residents of Hebei province in north China and were associated with SES conditions. This study indicated that from a public health perspective, more attention should be paid to screening of cardio-metabolic disorders in less developed areas.

## Introduction

Cardiovascular and metabolic diseases are leading causes of death and pose a great threat to public health, especially in developing countries (1–3). Metabolic syndrome (MetS) is a set of physiological and biochemical disorders that are characterized by pathological components such as abdominal obesity, impaired glucose metabolism, and increased blood pressure. It has been known that the occurrence of MetS is associated with increased risks of developing cardio-metabolic diseases such as diabetes, hypertension, coronary heart disease, and stroke (4). The prevalence of cardio-metabolic disorders is on the rise among Chinese populations and has already become the primary cause of death among Chinese residents (5).

Metabolic syndrome and its components are closely associated with increased risks of cardio-metabolic disorders (6, 7). Other cardio-metabolic risk factors include gender, lifestyle, family history of disease, and socioeconomic status (SES) (8). SES reflects a person’s position in society and mainly includes education, income, and occupation. Previous studies have demonstrated that lower SES was associated with higher risk of cardio-metabolic disorders in developed countries (9, 10). China has experienced great social and economic transitions in the last decades. These transitions accompany lifestyle changes such as abundance in high-calorie foods and decrease in physical work, and have led to sharp increase in cardio-metabolic diseases (11). Several studies have investigated the association between SES and prevalence of cardio-metabolic risk factors in China. However, most of these studies were carried out on developed areas (12, 13), and only few have been reported on populations in less developed areas. Hebei is a developing province located in the North China Plain and surrounded by the capital Beijing. This is the first time the association between SES and prevalence of cardio-metabolic risk factors among residents of Hebei province has been investigated.

## Materials and Methods

### Study Population

This epidemiological study was implemented in September 2016 and conducted in Hebei province. Sample selection was conducted based on a multistage, stratified sampling method. First, cities of Shijiazhuang and Renqiu were selected as representative urban and rural areas of Hebei province based on gross domestic product per capita. Second, one district was randomly selected from each city. Next, several residential communities were randomly selected from each district, and eligible individuals who met the inclusion criteria were included. The composition of age and sex of each community and urban-rural ratio are designed based on latest national census data (14) and are presented in Supplementary Table 1.

The inclusion criteria were as follows: age 18 years or older, has lived in the selected community for at least 5 years, and not pregnant. A total of 2,650 adults were randomly selected from local residents. Our study eventually included 2,638 participants after excluding 12 people with missing information on sex, age, plasma glucose, or SES questionnaire. This study was approved by the Medical Ethics Committee of The Second Hospital of Hebei Medical University, and all methods were performed in accordance with relevant guidelines and regulations. All participants provided written informed consent following a thorough explanation of research procedures. A flow diagram of the analytical sample is presented in Supplementary Figure 1.

### Clinical and Laboratory Measurements

A standardized questionnaire was administered by trained professionals to collect in regional locations. Body weight, height, waist circumference, and BP were measured by trained nurses according to standard protocols. Body mass index (BMI) was calculated as weight (kg)/height (m^2^) and was classified into three categories: normal (<24 kg/m^2^), overweight (≥24 and <28 kg/m^2^), and obese (≥28 kg/m^2^) according to the criteria (15). Waist circumference (WC) was measured midway between the lower border of the rib margin and the iliac crest at the end of normal expiration. Salt intake was classified into three categories: mild (<5 g/day), moderate (5–10 g/day), and severe (>10 g/day). Systolic blood pressure (SBP) and diastolic blood pressure (DBP) were measured with an electronic blood pressure monitor (Omron HEM-7430; Omron Corporation, Kyoto, Japan) on the non-dominant arm twice according to standard protocols.

After 12 h of overnight fasting, a blood sample was drawn from each participant, and fasting blood glucose (FBG), total cholesterol (TC), triglycerides (TGs), high-density lipoprotein cholesterol (HDL-C), and low-density lipoprotein cholesterol (LDL-C) were measured using an automatic biochemical analyzer (Mindray BS-180 Analyzer) according to standard protocols.

### Clinical Assessments

#### Cardio-Metabolic Risk Factors

The participants were diagnosed with MetS if they had at least three of the following criteria according to Chinese Diabetes Society-2017 (CDS-2017) (15): (1) abdominal obesity: WC ≥ 90 cm for males and WC ≥ 85cm for females; (2) elevated blood glucose (BG): ≥ 6.1 mmol/L or 2 h OGTT ≥ 7.8 mmol/L and (or) diabetes history; (3) elevated blood pressure (BP): ≥ 130/85 mmHg and (or) self-reported hypertension history; (4) elevated TG: ≥ 1.7 mmol/L; (5) Low HDL-C: < 1.04 mmol/L.

### Socioeconomic Status Assessment

In order to evaluate SES, variables were calculated using a score of 1–5. A socioeconomic score for a participant was the sum of the following three variables: education: (1) no formal education, (2) elementary school education, (3) junior middle school education, (4) senior high school education, and (5) university education and above; occupation: (1) no stable work, (2) labor work, (3) clerical work, (4) graduate/technician work, and (5) management positions; individual income per year: (1) less than 10,000 yuan, (2) 10,000–30,000 yuan, (3) 30,000–50,000 yuan, (4) 50,000–100,000 yuan, and (5) more than 100,000 yuan.

The composite score was calculated for every participant in SES dimension. The lowest composite score is 3 points and the highest is 15 points. The distribution of SES scores was spilt into tertiles where T1 was the reference group and T3 was the best.

### Statistical Analysis

To account for the complex sampling design of this study, we used the SUDAAN software (Research Triangle Institute, North Carolina, United States) to obtain estimates of prevalence and standard errors according to the Taylor linearization method. Estimates were weighted to reflect age, sex, and urban-rural distribution of adults living in Hebei province. Weighting coefficients were derived from the 2010 Chinese population census data, and the sampling scheme of our survey was designed to obtain a representative estimate. Categorical data presented as counts and percentages were analyzed by chi-square test or Fisher’s exact test, as appropriate. The POLYNOMIAL statement was used to assess significant linear trend and quadratic trend for the levels of an ordinal group. The normality of continuous variables was assessed, and variables with skewed distribution were reported with medians and interquartile ranges (IQRs); otherwise, continuous data are presented as means and standard deviations. Adjusted odds ratios (ORs) with 95% CIs were calculated by multivariable logistic regression to examine the association between independent variables with MetS and its components. Three models with progressively increased adjustment of risk factors among all the participants were applied for sensitive analysis (Supplementary Tables 2, 3). All statistical analyses were conducted using Statistical Analysis System version 9.3 (SAS Institute, Inc., North Carolina, NC, United States) and SUDAAN software version 10.0. A *p*-value of <0.05 was considered statistically significant.

## Results

### Basic Characteristics of the Study Population

Overall, 2,638 adults out of 2,650 invited local residents completed the questionnaire for this cross-sectional study, and 716 (27.1%) was diagnosed with MetS (Table 1). The prevalence of MetS was significantly higher in males (35.4%) than in females (18.4%) (*p* < 0.01). The prevalence of MetS increased with increase in age groups (Table 1). More than 90% of participants diagnosed with MetS were overweight or obese. People with MetS had significantly increased prevalence of smoking and heavy salt intake, and their SES conditions were also different compared with those with no MetS (all *p* < 0.05). There were significantly more under-educated (below senior high school) participants in the MetS (52.3%) group compared to the non-MetS (34.9%) group. Interestingly, we did not observe any difference in family income between participants in the MetS and non-MetS groups (*p* = 0.33).

**TABLE 1:** Baseline characteristics of the study by demographic data and clinical variables.

Characteristics	Total	MetS	Non-MetS	*P*-value
	(*n* = 2,638)	(*n* = 716)	(*n* = 1,922)	
Gender, *n* (%)				<0.01
Male	1352 (51.3)	479 (35.4)	873 (64.6)	
Female	1286 (48.7)	237 (18.4)	1049 (81.6)	
Location, *n* (%)				0.05
Urban area	1389 (52.7)	399 (28.7)	990 (71.3)	
Rural area	1249 (47.3)	317 (25.4)	932 (74.6)	
Age groups, *n* (%)				<0.01
18–29	673 (25.5)	67 (10.0)	606 (90.0)	
30–39	542 (20.5)	132 (24.4)	410 (75.6)	
40–49	576 (21.8)	144 (25.0)	432 (75.0)	
50–59	405 (15.4)	175 (43.2)	230 (56.8)	
60–69	248 (9.4)	108 (43.5)	140 (56.5)	
70–	194 (7.4)	90 (46.4)	104 (53.6)	
Smoking, *n* (%)				<0.01
No	1918 (72.7)	452 (23.6)	1466 (76.4)	
Yes	720 (27.3)	264 (36.7)	456 (63.3)	
Salt intake, *n* (%)				<0.01
Mild	420 (15.9)	96 (22.9)	324 (77.1)	
Moderate	1799 (68.2)	471 (26.2)	1328 (73.8)	
Heavy	419 (15.9)	149 (35.6)	270 (64.4)	
Family history of diabetes, *n* (%)				0.17
No	2,042 (77.4)	541 (26.5)	1,501 (73.5)	
Yes	596 (22.6)	175 (29.4)	421 (70.6)	
BMI, *n* (%)				<0.01
Normal	1,052 (39.9)	68 (64.6)	984 (35.4)	
Overweight	981 (37.2)	288 (29.4)	693 (70.6)	
Obesity	605 (22.9)	360 (59.5)	245 (40.5)	
Individual-level SES variables				
Education, *n* (%)				< 0.01
No formal education	138 (5.2)	57 (41.3)	81 (58.7)	
Elementary school education	228 (8.6)	105 (46.1)	123 (53.9)	
Junior middle school education	678 (25.7)	212 (31.3)	466 (68.7)	
Senior high school education	789 (29.9)	176 (22.3)	613 (77.7)	
University education and above	805 (30.5)	166 (20.6)	639 (79.4)	
Family income per year, *n* (%)				0.33
Less than 10,000 CNY	651 (24.7)	188 (28.9)	463 (71.1)	
10,000–30,000 CNY	474 (18.0)	120 (25.3)	354 (74.7)	
30,000–50,000 CNY	726 (27.5)	199 (27.4)	527 (72.6)	
50,000–100,000 CNY	620 (23.5)	173 (27.9)	447 (72.1)	
More than 100,000 CNY	167 (6.3)	36 (21.6)	131 (78.4)	
Occupation, *n* (%)				<0.01
No stable work	895 (33.9)	215 (24.0)	680 (76.0)	
Labor work	1204 (45.6)	378 (31.4)	826 (68.6)	
Clerical work	487 (18.5)	111 (22.8)	376 (77.2)	
Graduate/technician work	47 (1.8)	10 (21.3)	37 (78.7)	
Management positions	5 (0.2)	2 (40.0)	3 (60.0)	
SES groups, *n* (%)				<0.01
T1	818 (31.0)	269 (32.9)	549 (67.1)	
T2	946 (35.9)	240 (25.4)	706 (74.6)	
T3	874 (33.1)	207 (23.7)	667 (76.3)	
**Clinical variables, mean ± SD**				
Age, years	42.55 ± 16.08	50.19 ± 14.89	39.70 ± 15.57	<0.01
BMI, kg/m^2^	25.15 ± 4.02	28.21 ± 3.46	24.01 ± 3.60	<0.01
WC, cm	85.03 ± 11.20	94.91 ± 8.00	81.35 ± 9.93	<0.01
SBP, mmHg	127.30 ± 19.50	140.84 ± 18.26	122.25 ± 17.43	<0.01
DBP, mmHg	79.50 ± 11.90	87.32 ± 10.87	76.59 ± 10.92	<0.01
FBG, mmol/L	5.62 ± 1.50	6.47 ± 2.19	5.31 ± 0.96	<0.01
TG, mmol/L	1.63 ± 1.48	2.67 ± 2.12	1.24 ± 0.88	<0.01
TC, mmol/L	4.63 ± 1.10	5.07 ± 1.08	4.46 ± 1.06	<0.01
HDL-C, mmol/L	1.39 ± 0.37	1.17 ± 0.31	1.47 ± 0.36	<0.01
LDL-C, mmol/L	2.63 ± 0.79	2.95 ± 0.80	2.52 ± 0.75	<0.01

*MetS, metabolic syndrome; BMI, body mass index; SES, socioeconomic status; T, tertile; WC, waist circumference; SBP, systolic blood pressure; DBP, diastolic blood pressure; FBG, fasting blood glucose; TG, triglycerides; TC, total cholesterol; HDL-C, high density lipoprotein-cholesterol; LDL-C, low density lipoprotein-cholesterol; CNY, Chinese Yuan.*

Compared with those with no MetS, participants with MetS reported significantly higher levels of age, BMI, and WC, and higher values of SBP, DBP, FBG, TG, TC, and LDL-C, and lower values of HDL-C (all *p* < 0.01). Meanwhile, we found no significant differences in terms of location and family history of diabetes in the MetS and non-MetS groups (*p* > 0.05).

### Weighted Prevalence of MetS and Its Components Across Groups of SES

The most common cardio-metabolic risk factors were elevated BP (46.2%), abdominal obesity (42.9%), elevated TG (31.2%), elevated BG (23.4%), and low HDL-C (14.9%). As shown in Table 2, the weighted prevalence of MetS (26.8%) and its components were different under three SES conditions. In general, a negative linear association was found between prevalence of MetS and abdominal obesity with increase in SES groups.

**TABLE 2:** Weighted prevalence of MetS and its components across groups of SES.

Diagnosis, *n* (%)	SES groups				*P* for difference	*P* for linear trend	*P* for quadratic trend
	T1 (*n* = 818)	T2 (*n* = 946)	T3 (*n* = 874)	Total (*n* = 2,638)			
MetS	269 (33.7%)	240 (25.1%)	207 (22.2%)	716 (26.8%)	0.01	0.01	0.65
Abdominal obesity	405 (53.6%)	392 (41.9%)	309 (34.2%)	1106 (42.9%)	0.04	0.04	0.79
Elevated BG	269 (33.2%)	204 (20.5%)	171 (17.4%)	644 (23.4%)	0.13	0.09	0.38
Elevated BP	472 (59.3%)	424 (46.4%)	308 (33.8%)	1204 (46.2%)	0.11	0.14	0.99
Elevated TG	240 (28.9%)	308 (32.0%)	308 (32.4%)	856 (31.2%)	0.08	0.38	0.44
Low HDL-C	110 (14.7%)	131 (13.6%)	144 (16.5%)	385 (14.9%)	0.66	0.27	0.21

*T, tertile; MetS, metabolic syndrome; BG, blood glucose; BP, blood pressure; TG, triglycerides; HDL-C, high density lipoprotein-cholesterol.*

### Adjusted ORs for MetS and Its Components Among Different Risk Factors

Multivariate logistic regression analysis suggested that the associations between SES levels and MetS were not statistically significant. Participants with higher SES condition (T3) had a significantly lower risk of having elevated BP (OR:0.71, 95% CI:0.54–0.92) than those with lower SES (T1). The analysis also suggested that age was an important risk factor for MetS and its components, with ORs appearing an increasing trend with increase in age, except for elevated TG and low HDL-C. In addition, the ORs of MetS (OR:0.48; 95% CI:0.37–0.61), abdominal obesity (OR:0.43; 95% CI:0.34–0.56), elevated BG (OR:0.78; 95% CI:0.62–0.99), elevated BP (OR:0.46; 95% CI:0.37–0.57), elevated TG (OR:0.76; 95% CI:0.61–0.94), and low HDL-C (OR:0.38; 95% CI:0.28–0.51) were significantly decreased in females than males. The ORs of MetS and its components increased significantly in participants from overweight to obesity. Participants living in rural areas had a lower risk of developing elevated BG and elevated TG but have an increased risk of abdominal obesity, elevated BP, and low HDL-C compared with people living in urban areas. Our data also suggested that heavy salt intake, smoking, and diabetes history were associated with the prevalence of cardio-metabolic parameters.

Adjusted ORs for MetS and its components between SES in males and females are presented in Table 4. It is observed that females with higher SES had significantly lower ORs in developing elevated BP (OR:0.65, 95% CI:0.43–0.97). Adjusted ORs for MetS and its components between SES in urban or rural areas are presented in Table 5. The data showed that people with higher SES had a high risk of developing elevated BG (OR: 1.67, 95% CI: 1.08–2.59) in urban areas and low risk of having an elevated BP (OR:0.56, 95% CI:0.38–0.84) in rural areas.

Unadjusted and age-sex-adjusted odds ratios for MetS and its components among risk factors by sensitivity analysis are presented in Supplementary Tables 2, 3.

## Discussion

Metabolic syndrome (MetS) has become a major threat to public health worldwide (16). If left untreated, patients with MetS are disposed to a series of chronic diseases such as diabetes, cardiovascular disease, and cognitive dysfunction, and have increased all-cause and cardiovascular death (17, 18).

Among all diagnosing guidelines, Adult Treatment Panel III (ATP III- 2002) (19), International Diabetes Federation criteria (IDF-2004) (20), and Chinese Diabetes Society (CDS-2004) (21) are three frequently used guidelines in clinical practice and published articles. However, the diagnostic results are largely affected by which one of these three guidelines are applied in the analysis (22). Given that the Chinese have experienced rapid socioeconomic transitions during these years, adopting the latest CDS criteria (CDS-2017) might increase the accuracy of describing the prevalence of MetS in Chinese adults (15).

The prevalence of MetS has been increasing in recent years (16), especially in China, which faced great social and economic transitions in the last decade. Identifying populations at risk would contribute to more effective screening and prevention. Generally, the prevalence of MetS varies in different regions in China. It was reported that the adjusted prevalence of MetS across China was 11% in 2010 by 2004 CDS criteria (23). The prevalence of MetS was 16.7% in adult Hong Kong Chinese in 2005 by NCEP ATP III (24). In highly urbanized Beijing, MetS prevalence was 14.05% for males and 28.55% for females according to NCEP ATP III in 2007 (25). In our study, the weighted prevalence of MetS was 26.8% in Hebei, and 18.4% for females and 35.4% for males. Possible origins may be attributed to inequality in education, income, and occupation, and their associations with health between developed and developing areas in China, which has been discussed in many bodies of literature (26, 27).

A composite SES score was then calculated to better understand the overall condition of SES (28). Patients with lower SES condition had increased prevalence of developing cardio-metabolic disorders, such as MetS and abdominal obesity (Table 2). Our results were consistent with earlier studies, which demonstrated that better SES is associated with lower rates of specific diseases such as coronary disease, diabetes, cognitive impairment, stroke, cancer, and arthritis (29, 30). Higher SES is generally associated with better education, income, medical services and other social benefits that provide positive effects on health (31). However, economic stress and social vulnerability are risk factors for various chronic disorders, particularly cardiovascular diseases (17). People with lower SES are generally more stressed, which often leads to restricted opportunities for good health services (32).

Differences in prevalence of MetS between males (35.4%) and females (18.4%) were significant (*p* < 0.01). Consistent with a previous study, our data suggested that females were less likely to develop MetS and other related cardio-metabolic risk parameters (Table 3) (33). After multivariate adjustments, the ORs for MetS increased with higher SES levels in males. As for females, inverse associations were shown between SES and MetS. Lower SES was associated with higher risk of MetS among females (Table 4). However, the associations between them were not statistically significant, which might be attributed to lack of sufficient sample size. This was consistent with other findings in Korea (34) and China (25). Another possible explanation may contribute to the gender differences. Males with better SES may spend more time sitting in the office, have little time to exercise, frequently consume high-fat foods, and suffer from work-related mental health problems (35).

**TABLE 3:** Associations of independent variables with MetS and its components in multivariate logistic regression analysis.

Variables	MetS	Abdominal obesity	Elevated BG	Elevated BP	Elevated TG	Low HDL-C
	OR (95% CI)	OR (95% CI)	OR (95% CI)	OR (95% CI)	OR (95% CI)	OR (95%CI)
**SES**						
T1	Reference	Reference	Reference	Reference	Reference	Reference
T2	0.87 (0.66, 1.15)	0.83 (0.62, 1.10)	1.04 (0.79, 1.37)	0.87 (0.69, 1.10)	1.05 (0.82, 1.34)	0.76 (0.55, 1.05)
T3	0.96 (0.70, 1.33)	0.79 (0.57, 1.09)	1.10 (0.80, 1.51)	0.71 (0.54, 0.92)*	1.07 (0.81, 1.41)	1.09 (0.77, 1.55)
**Age**						
18–29	Reference	Reference	Reference	Reference	Reference	Reference
30–39	2.06 (1.43, 2.97)*	1.91 (1.35, 2.71)*	3.47 (2.23, 5.40)*	1.58 (1.202, 2.08)*	2.24 (1.68, 2.98)*	0.98 (0.69, 1.31)
40–49	2.21 (1.55, 3.17)*	1.87 (1.33, 2.62)*	5.68 (3.71, 8.71)*	2.17 (1.66, 2.83)*	2.32 (1.75, 3.07)*	0.75 (0.52, 1.07)
50–59	5.93 (4.09, 8.60)*	5.55 (3.86, 7.98)*	12.57 (8.16, 19.38)*	4.51 (3.37, 6.05)*	2.60 (1.92, 3.53)*	0.85 (0.58, 1.25)
60–69	6.24 (4.06, 9.59)*	4.30 (2.81, 6.58)*	20.17 (12.49, 32.57)*	5.88 (4.089, 8.46)*	2.06 (1.41, 2.99)*	0.72 (0.44, 1.18)
70–	8.74 (5.46, 13.98)*	5.31 (3.34, 8.42)*	36.18 (21.65, 60.46)*	9.37 (6.10, 14.38)*	2.00 (1.31, 3.03)*	0.73 (0.42, 1.28)
**Sex**						
Male	Reference	Reference	Reference	Reference	Reference	Reference
Female	0.48 (0.37, 0.61)*	0.43 (0.34, 0.56)*	0.78 (0.62, 0.99)*	0.46 (0.37, 0.57)*	0.76 (0.61, 0.94)*	0.38 (0.28, 0.51)*
**BMI**						
Normal	Reference	Reference	Reference	Reference	Reference	Reference
Overweight	4.28 (3.19, 5.76)*	6.80 (5.26, 8.80)*	1.49 (1.16, 1.92)*	2.18 (1.77, 2.68)*	2.36 (1.89, 2.95)*	3.85 (2.72, 5.44)*
Obesity	18.26 (13.36, 24.96)*	85.50 (59.93, 121.97)*	3.59 (2.73, 4.71)*	4.83 (3.80, 6.14)*	4.24 (3.33, 5.40)*	7.08 (4.97, 10.08)*
**Area**						
Urban	Reference	Reference	Reference	Reference	Reference	Reference
Rural	0.95 (0.77, 1.19)	1.61 (1.28, 2.02)*	0.75 (0.60, 0.93)*	1.23 (1.02, 1.48)*	0.45 (0.37, 0.55)*	1.66 (1.30, 2.13)*
**Smoking**						
No	Reference	Reference	Reference	Reference	Reference	Reference
Yes	1.31 (1.02, 1.68)*	0.99 (0.76, 1.30)	1.10 (0.85, 1.42)	0.93 (0.74, 1.16)	1.42 (1.14, 1.78)*	1.35 (1.04, 1.77)*
**Salt intake**						
Mild	Reference	Reference	Reference	Reference	Reference	Reference
Moderate	1.22 (0.91, 1.66)	1.09 (0.80, 1.47)	0.89 (0.67, 1.18)	0.85 (0.66, 1.09)	1.13 (0.88, 1.45)	0.96 (0.68, 1.36)
Heavy	1.34 (0.93, 1.93)	1.03 (0.71, 1.50)	0.87 (0.61, 1.23)	1.04 (0.76, 1.43)	1.51 (1.10, 2.06)*	1.13 (0.75, 1.71)
**Family history of diabetes**						
No	Reference	Reference	Reference	Reference	Reference	Reference
Yes	1.37 (1.07, 1.75)*	0.98 (0.76, 1.26)	1.66 (1.30, 2.11)*	0.87 (0.70, 1.07)	1.28 (1.03, 1.58)*	1.766 (1.36, 2.30)*

*Adjusted for age, sex, BMI, SES, area, smoking, salt intake, and family history of diabetes. BMI, Body Mass Index; SES:Socioeconomic status; T, Tertile; MetS, Metabolic Syndrome; BG, blood Glucose; BP, Blood Pressure; TG, triglycerides; HDL-C, High-Density Lipoprotein-Cholesterol. *Statistically significant.

**TABLE 4:** Adjusted odds ratios in SES for MetS and its components between risk factors in males or females.

Variables	MetS	Abdominal obesity	Elevated BG	Elevated BP	Elevated TG	Low HDL-C
	OR (95% CI)	OR (95% CI)	OR (95% CI)	OR (95% CI)	OR (95% CI)	OR (95% CI)
**SES in males**						
T1	Reference	Reference	Reference	Reference	Reference	Reference
T2	1.04 (0.71, 1.51)	0.93 (0.62, 1.39)	1.28 (0.89, 1.85)	0.90 (0.64, 1.27)	1.06 (0.75, 1.51)	0.67 (0.43, 1.04)
T3	1.19 (0.79, 1.80)	0.85 (0.54, 1.33)	0.99 (0.67, 1.47)	0.76 (0.53, 1.10)	1.39 (0.95, 2.04)	1.07 (0.67, 1.69)
**SES in females**						
T1	Reference	Reference	Reference	Reference	Reference	Reference
T2	0.87 (0.59, 1.28)	0.77 (0.51, 1.17)	0.78 (0.57, 1.08)	0.84 (0.60, 1.18)	1.08 (0.76, 1.53)	0.90 (0.65, 1.25)
T3	0.72 (0.45, 1.15)	0.80 (0.48, 1.33)	0.81 (0.57, 1.15)	0.65 (0.43, 0.97)*	0.73 (0.48, 1.10)	1.15 (0.79, 1.66)

*Adjusted for age, BMI, SES, area, smoking, salt intake, and family history of diabetes. BMI, body mass index; SES, socioeconomic status; T, tertile; MetS, metabolic syndrome; BG, blood glucose; BP, blood pressure; TG, triglycerides; HDL-C, high-density lipoprotein-cholesterol. *Statistically significant.

The analysis was then stratified by living area (Table 5). The ORs for elevated BG increased with higher SES levels in urban areas and for elevated BP decreased with higher SES levels in rural areas. The possible mechanism for different regional impacts of SES on MetS in urbanization may through unhealthy behaviors, such as decreased physical activity, excessive intake of animal fats and salts, and low intake of fruits and vegetables in urban areas (36).

**TABLE 5:** Adjusted odds ratios in SES for MetS and its components between risk factors in urban or rural area.*

Variables	MetS	Abdominal obesity	Elevated BG	Elevated BP	Elevated TG	Low HDL-C
	OR (95% CI)	OR (95% CI)	OR (95% CI)	OR (95% CI)	OR (95% CI)	OR (95% CI)
**SES in urban area**						
T1	Reference	Reference	Reference	Reference	Reference	Reference
T2	0.94 (0.61, 1.45)	0.68 (0.44, 1.04)	1.33 (0.88, 2.01)	0.792 (0.543, 1.153)	1.21 (0.85, 1.73)	0.96 (0.56, 1.64)
T3	1.18 (0.75, 1.85)	0.76 (0.49, 1.19)	1.67 (1.08, 2.59)*	0.78 (0.532, 1.144)	1.12 (0.78, 1.62)	1.31 (0.77, 2.23)
**SES in rural area**						
T1	Reference	Reference	Reference	Reference	Reference	Reference
T2	0.83 (0.57, 1.21)	0.94 (0.63, 1.40)	0.85 (0.58, 1.25)	0.99 (0.72, 1.35)	0.90 (0.63, 1.28)	0.66 (0.43, 1.01)
T3	0.74 (0.45, 1.21)	0.78 (0.47, 1.31)	0.61 (0.35, 1.05)	0.56 (0.38, 0.84)*	1.02 (0.65, 1.58)	1.01 (0.62, 1.66)

*Adjusted for age, sex, BMI, SES, area, smoking, salt intake, and family history of diabetes. BMI, body mass index; SES, socioeconomic status; T, tertile; MetS, metabolic syndrome; BG, blood glucose; BP, blood pressure; TG, triglycerides; HDL-C, high-density lipoprotein-cholesterol. *Statistically significant.

Moreover, a relationship was observed between prevalence of MetS and age in both males and females, which is similar to other studies (37, 38). The increased prevalence of MetS with age can be attributed to similar age-related trends in all components of MetS (39, 40). The most common cardio-metabolic risk factors in Hebei province we identified in this study were elevated BP (45.6%) and abdominal obesity (42.9%). These data raised a red flag to public health that urgent measures need to be taken to prevent elevated BP-related and obesity-related cardio-metabolic disorders in high-risk populations of Hebei province. Additionally, smoking and family history of diabetes were also independent risk factors for MetS and its components, as seen in previous studies (41).

Previous studies have shown that the link between SES and MetS was significant and positive (28). However, our results found that the association between them was not statistically significant (Table 3), which was consistent with other studies (42, 43). Several explanations are possible. First, despite several diagnostic criteria of MetS such as ATP III criteria, IDF criteria, and CDS criteria, worldwide-accepted criteria do not exist. The CDS-2017 criteria were used in our study for better applicability in Chinese populations. Second, there is no unique definition for SES. It can be social, economic, or psychosocial. An analysis per type of status could show different links with MetS in terms of significance, sign, and magnitude. Third, our study focused mainly on age, sex, and living area. Other behavioral risk factors such as alcohol use, dietary quality, and physical activity were not discussed. Fourth, the null association between SES and MetS may be due to sample size issues. Our study only included data from a region in north China. The conclusion of this study should be further examined in prospective studies in the future.

In summary, the prevalence of MetS and cardio-metabolic risk factors is partially conditioned by individual SES status, which should be taken into account by healthcare professionals when making preventive strategies to reduce health inequality in society. Overall, our results supported the idea that there was a definite association between SES and prevalence of cardio-metabolic risk factors in a developing area in China.

## Conclusion

This was the first cross-sectional study to examine the associations between SES and MetS, as well as its cardio-metabolic components, in north China, Hebei province. Our data suggested that better SES conditions were associated with lower prevalence of MetS and abdominal obesity. Lower SES condition, male gender, older age, obesity, smoking, and family history of diabetes were associated with higher risk of developing cardio-metabolic disorders. This should be the target group for possible early lifestyle intervention to reduce the occurrence of cardio-metabolic disorders in less developed areas in China.

## Data Availability Statement

The original contributions presented in the study are included in the article/Supplementary Material, further inquiries can be directed to the corresponding author.

## Ethics Statement

The studies involving human participants were reviewed and approved by Medical Ethics Committee of The Second Hospital of Hebei Medical University. The patients/participants provided their written informed consent to participate in this study.

## Author Contributions

ZH analyzed the data, drafted, reviewed, and edited the manuscript, and contributed to the discussion. LZ and MW conducted, designed, and supervised the study, reviewed and edited the manuscript, and contributed to the discussion. QZ, JL, ZL, LY, and YZ complemented the study, reviewed the manuscript and contributed to the discussion. All authors read and approved the final version of the manuscript.

## Conflict of Interest

The authors declare that the research was conducted in the absence of any commercial or financial relationships that could be construed as a potential conflict of interest.

## Publisher’s Note

All claims expressed in this article are solely those of the authors and do not necessarily represent those of their affiliated organizations, or those of the publisher, the editors and the reviewers. Any product that may be evaluated in this article, or claim that may be made by its manufacturer, is not guaranteed or endorsed by the publisher.
