# Supplementary figures and images for: Association Between Socioeconomic Status and Prevalence of Cardio-Metabolic Risk Factors: A Cross-Sectional Study on Residents in North China
Source: Front Cardiovasc Med. 2022 Mar 7;9:698895. doi: 10.3389/fcvm.2022.698895 (PMC8940519; doi:10.3389/fcvm.2022.698895)

**Supplementary figure 1. Flow diagram of analytical sample.**

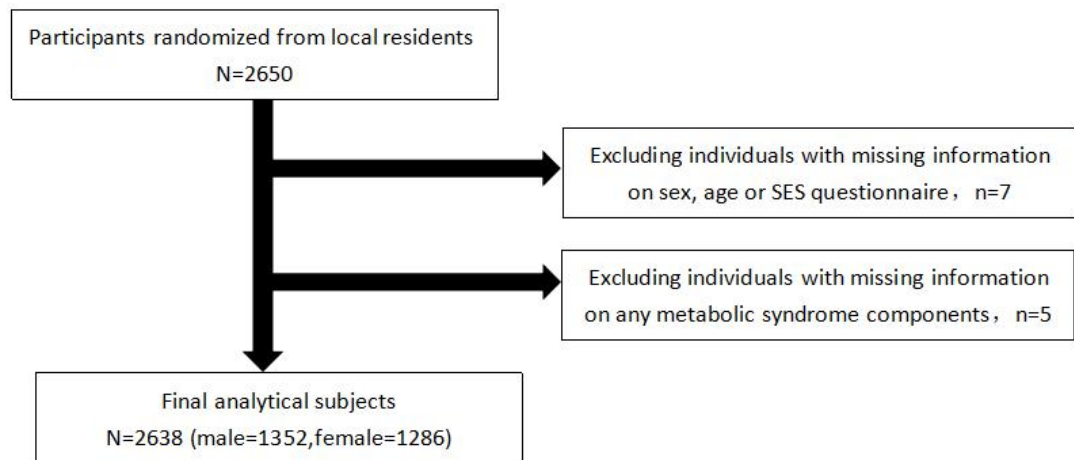

Supplement: Supplementary file 1 [file Image_1.pdf]
