# Supplementary material for: Association Between Socioeconomic Status and Prevalence of Cardio-Metabolic Risk Factors: A Cross-Sectional Study on Residents in North China
Source: Front Cardiovasc Med. 2022 Mar 7;9:698895. doi: 10.3389/fcvm.2022.698895 (PMC8940519; doi:10.3389/fcvm.2022.698895)
Supplement: Supplementary file 2 [file Table_1.pdf]

Supplementary table 1. The designed composition of age and sex of each community and urban-rural ratio based on the latest national census data.

Age	Sample n(%)	Urban area (n=1393)		Rural area (n=1257)	
		Male	Female	Male	Female
18-29	676（25.51）	184	172	165	155
30-39	547（20.64）	145	140	133	129
40-49	577（21.77）	157	149	138	133
50-59	406（15.33）	108	104	101	93
≥60	444（16.75）	121	113	105	105
Total	2650（100）	715	678	642	615
