# Supplementary material for: Association Between Socioeconomic Status and Prevalence of Cardio-Metabolic Risk Factors: A Cross-Sectional Study on Residents in North China
Source: Front Cardiovasc Med. 2022 Mar 7;9:698895. doi: 10.3389/fcvm.2022.698895 (PMC8940519; doi:10.3389/fcvm.2022.698895)
Supplement: Supplementary file 3 [file Table_2.pdf]

Supplementary table 2. Diagnostic criteria of metabolic disorders.

		Diagnostic criteria	Source of diagnostic criteria
MetS	More than 3 of the following criteria were apparent in a participant	(1) waist circumference: male ≥90 cm, women ≥85 cm;<br>(2) TG ≥1.7 mmol/L;<br>(3) HDL-C level: males <1.0 mmol/L, females <1.3 mmol/L;<br>(4) abnormal blood pressure: presence of any of the following 3 criteria: (a) taking antihypertensive medications;<br>(b) SBP ≥130 mmHg; (c) DBP ≥85 mmHg;<br>(5) hyperglycemia: any of the following 4 criteria related to blood glucose: (a) self-reported diabetes history or taking hypoglycemic drugs;<br>(b) FPG ≥5.6 mmol/L;<br>(c) OGTT-2hPG ≥7.8 mmol/L;<br>(d) HBA1C ≥5.7%.	International Diabetes Federation (IDF) in 2004.[9]
Diabetes	One or >1 of the following criteria were present	(1) FPG ≥7·0 mmol/L;<br>(2) OGTT-2hPG ≥11.1 mmol/L;<br>(3) HBA1C ≥6.5%.	American Diabetes Association (ADA) in 2018.[10]
Prediabetes		(1) FPG: 5.6–7.0 mmol/L;<br>(2) OGTT-2hPG 7.8–<11.1 mmol/L;<br>(3) HBA1C 5.7%–6.5%;<br>(4) non-diabetic.	
Hypertention		(1) taking antihypertensive medications;<br>(2) SBP ≥140 mmHg;<br>(3) DBP ≥90 mmHg;	
Central obesity		Waist circumference:<br>male ≥90 cm,<br>women ≥85 cm;	Bao Y, et al. *Atherosclerosis*. 2008.[11]
Hypertriglyceridemia		TG ≥1.7 mmol/L	*Circulation*· 2002.[12]
Hypercholesterolemia		TC ≥5.2 mmol/L	
High LDL-C		LDL-C ≥3.4 mmol/L	

Low HDL-C	Male: HDL-C <1.0 mmol/L<br>Female：HDL-C <1.3 mmol/L
Metabolic disorder	Satisfied any of diseases above
