# Supplementary material for: Association Between Socioeconomic Status and Prevalence of Cardio-Metabolic Risk Factors: A Cross-Sectional Study on Residents in North China
Source: Front Cardiovasc Med. 2022 Mar 7;9:698895. doi: 10.3389/fcvm.2022.698895 (PMC8940519; doi:10.3389/fcvm.2022.698895)
Supplement: Supplementary file 4 [file Table_3.pdf]

Supplementary table 3. Comparison in the prevalence of metabolic disorders between TIDE and Hebei.

	Total		Men		Women	
Diagnosis	TIDE	Hebei	TIDE	Hebei	TIDE	Hebei
Metabolic disorders	81.2%	84.8%	85.0%	89.3%	76.7%	80.0%
MetS	28.6%	36.8%	32.5%	43.6%	24.1%	29.7%
Diabetes	8.7%	14.2%	9.3%	17.3%	8.0%	11.0%
Prediabetes	39.4%	42.5%	39.1%	44.8%	34.7%	39.2%
Hypertention	29.3%	31.5%	34.4%	38.2%	23.5%	24.5%
Central obesity	34.5%	41.9%	39.0%	52.1%	29.3%	31.3%
Hypertriglyceridemia	28.5%	32.4%	35.0%	38.6%	21.0%	26.0%
Hypercholesterolemia	28.2%	27.1%	28.7%	26.4%	27.7%	27.8%
High LDL-C	21.1%	15.0%	23.4%	15.8%	18.5%	14.3%
Low HDL-C	19.5%	22.8%	13.9%	16.1%	26.1%	29.8%
